# Physico-Chemical Evaluation of Rationally Designed Melanins as Novel Nature-Inspired Radioprotectors

**DOI:** 10.1371/journal.pone.0007229

**Published:** 2009-09-30

**Authors:** Andrew D. Schweitzer, Robertha C. Howell, Zewei Jiang, Ruth A. Bryan, Gary Gerfen, Chin-Cheng Chen, Dennis Mah, Sean Cahill, Arturo Casadevall, Ekaterina Dadachova

**Affiliations:** 1 Department of Nuclear Medicine, Albert Einstein College of Medicine, New York, New York, United States of America; 2 Howard Hughes Medical Institute, Medical Fellows Program, Chevy Chase, Maryland, United States of America; 3 The Mount Sinai School of Medicine, New York, New York, United States of America; 4 Department of Physiology and Biophysics, Albert Einstein College of Medicine, New York, New York, United States of America; 5 Department of Radiation Oncology, Albert Einstein College of Medicine, New York, New York, United States of America; 6 Department of Biochemistry, Albert Einstein College of Medicine, New York, New York, United States of America; 7 Department of Microbiology and Immunology, Albert Einstein College of Medicine, New York, New York, United States of America; 8 Department of Medicine, Albert Einstein College of Medicine, New York, New York, United States of America; Hebrew University of Jerusalem, Israel and University of California at Berkeley, United States of America

## Abstract

**Background:**

Melanin, a high-molecular weight pigment that is ubiquitous in nature, protects melanized microorganisms against high doses of ionizing radiation. However, the physics of melanin interaction with ionizing radiation is unknown.

**Methodology/Principal Findings:**

We rationally designed melanins from either 5-S-cysteinyl-DOPA, L-cysteine/L-DOPA, or L-DOPA with diverse structures as shown by elemental analysis and HPLC. Sulfur-containing melanins had higher predicted attenuation coefficients than non-sulfur-containing melanins. All synthetic melanins displayed strong electron paramagnetic resonance (2.14·10^18^, 7.09·10^18^, and 9.05·10^17^ spins/g, respectively), with sulfur-containing melanins demonstrating more complex spectra and higher numbers of stable free radicals. There was no change in the quality or quantity of the stable free radicals after high-dose (30,000 cGy), high-energy (^137^Cs, 661.6 keV) irradiation, indicating a high degree of radical stability as well as a robust resistance to the ionizing effects of gamma irradiation. The rationally designed melanins protected mammalian cells against ionizing radiation of different energies.

**Conclusions/Significance:**

We propose that due to melanin's numerous aromatic oligomers containing multiple π-electron system, a generated Compton recoil electron gradually loses energy while passing through the pigment, until its energy is sufficiently low that it can be trapped by stable free radicals present in the pigment. Controlled dissipation of high-energy recoil electrons by melanin prevents secondary ionizations and the generation of damaging free radical species.

## Introduction

Life on Earth exits in the constant flux of both ionizing and non-ionizing electromagnetic radiation. Consequently, various strategies to protect living organisms against radiation insults have emerged during evolution. Melanin, a high molecular weight pigment that is ubiquitous in nature, has been described to function as a free radical scavenger and has characteristics of an amorphous semiconductor [1]. Many microorganisms constitutively synthesize melanin including human pathogenic fungi *Cryptococcus neoformans* and *Histoplasma capsulatum*, and this pigment is known to protect these fungi against oxidants, extremes in temperature, UV light, chemotherapeutic drugs, and microbicidal peptides [2]. The ability of free-living microorganisms to make melanin is likely to be associated with a survival advantage in the environment [3] that includes protection against solar [4] and ionizing radiation [5]. Dramatic examples of such radiation protection are provided by the reports that melanized microorganisms are colonizing the highly radioactive environment inside the damaged nuclear reactor in Chernobyl [6] and cooling pool water in nuclear reactors [7].

One in two cancer patients is treated with radiation therapy at some point in the course of their disease. The availability of radioprotective materials suitable for internal administration which would protect normal organs without protecting the tumor would greatly enhance the efficacy of radiation therapy by permitting higher tumoricidal doses while protecting normal organs. Radioprotective materials could also be extremely useful for protection against terrorist actions using radiological devices and for protecting astronauts in space. This highlights the enormous need for novel nature-inspired radioprotectors.

Although there is an ample body of literature describing the optical, condensed phase electric, electron exchange, paramagnetic, UV-visible absorbance, and ion exchange properties of melanin [8], the mechanism of melanin's interaction with ionizing radiation remains relatively unexplored. We recently provided evidence for the capacity of melanins to function in transducing the energy of gamma radiation in living cells [9] and that the radioprotective efficacy of fungal melanins is dependent on their chemical composition [10]. Our goal is to rationally design, and evaluate by a battery of physico-chemical methods the melanins as novel radioprotectors for internal administration. For that purpose, we needed well-controlled conditions of chemical synthesis to yield pure melanins, as fungal melanins contain cell wall polysaccharides in their structure [11], and thus might be harmful or immunogenic if used for radiation shielding in humans.

Free radical scavenging, primarily of the highly destructive, short-lived radicals generated by the radiolysis of water, has been assumed to be the mechanism of melanin radioprotection [12]. However, the actual mechanism of melanin's interaction with ionizing radiation remains unexplored. We hypothesize that the physical interaction between melanin and the recoil electrons generated by Compton scattering of incident photons in melanin itself or transferred to melanin by other molecules and radicals is another significant contributor to the mechanism of radioprotection. A single Compton recoil electron traveling through tissue can result in multiple points of damage to DNA or other cell structures via the generation and propagation of free radical species [13]. We hypothesize that, due to melanin's numerous aromatic oligomers containing multiple π-electron systems, a generated Compton recoil electron gradually loses energy while passing through the pigment, until its energy is sufficiently low that it can be trapped by stable free radicals present in the pigment. Controlled dissipation of high-energy recoil electrons by melanin would prevent secondary ionizations and the generation of free radical species.

To test this hypothesis we synthesized chemically diverse melanins using precursors with different functional groups. To select melanins with the best radioprotective properties from the panel of synthesized melanins, we determined their physico-chemical characteristics to identify those that correlated with favorable radioprotective properties. Consequently, the melanins were subjected to: (i) elemental analysis, (ii) high performance liquid chromatography (HPLC), and (iii) electron paramagnetic resonance (EPR). Surrogate measurements of radioprotective properties were obtained by performing: (i) quantitative EPR to estimate the number of stable free radicals, (ii) determination of mass attenuation coefficients from the cross section used in Monte Carlo simulation; and (iii) clonogenic survival assay to determine melanin's ability to protect mammalian cells.

## Methods

### Melanin synthesis

Benzoyl peroxide dopamine melanin (MELex5) was synthesized using a method adapted from Gallas [14]. 5.0 g of dopamine and 1.2 ml of triethylamine was added to 100 ml of chloroform. 2.5 g of benzoyl peroxide was added to the reaction mixture and incubated at room temperature for 24 hours. The resulting mixture was vacuum-filtered; the residue was discarded and the filtrate was evaporated to dryness. The resulting solid was washed once with 40 ml of 70% ethanol, once with 40 ml 1% acetic acid, once with 40 ml of de-ionized water, and then lyophilized.

Tyrosinase melanins were synthesized using identical precursor ratios as Ito and Fujita [15] with only minor modifications in the method of synthesis. Melanin precursors were incubated with constant shaking at 37°C overnight with 8300 units of mushroom tyrosinase (Sigma) in 40 ml of 0.05 M sodium phosphate buffer, pH 6.8. The precursor for tyrosinase dopamine melanin (MEL1) was 0.5 mmol dopamine. The precursor for tyrosinase L-DOPA melanin (MEL2) was 0.5 mmol L-DOPA. The precursors for tyrosinase DOPA/Cys melanin (MEL3b) were 0.5 mmol L-DOPA and 0.75 mmol L-cysteine. The precursor for tyrosinase 5-S-CD melanin (MEL4) was 0.5 mmol 5-S-cysteinyldopa with 0.025 mmol L-DOPA added as a catalyst. After incubation overnight, the oxidation reaction was stopped with the addition of 250 µl of 6 M HCl (to pH ca. 3.0). The acidified mixture was kept at 2°C for 1 h. The precipitate was collected by centrifugation, washed three times with 15 ml of 1% acetic acid, washed twice with 15 ml acetone, once more with 15 ml of 1% acetic acid, and re-suspended in de-ionized water. The melanins were then lyophilized. Elemental analysis was performed by Quantitative Technologies, Inc. (Whitehouse, NJ).

### Permanganate oxidation of synthetic melanins

Oxidation was performed as in [15] with minor modifications. 0.5 mg of melanin was suspended in 1 ml of 1 M H_2_SO_4_, and 20 µl portions of 3% KMnO_4_ were added to the mixture. Each addition of KMnO_4_ was made immediately after its purple color had disappeared, and was followed immediately with vigorous vortexing. The oxidation reaction was stopped 10 min after the first KMnO_4_ addition by the addition of 100 µl of 10% Na_2_SO_3_. The resulting solution was centrifuged and an aliquot of 10 to 50 µl of the supernatant was used for each run of HPLC.

### Peroxide oxidation of synthetic melanins

Oxidation was performed as in [16] with minor modifications. 0.1 mg of melanin was suspended in 100 µl of deionized water. 860 µl of 1 M K_2_CO_3_ and 40 µl of 3% H_2_O_2_ were added to the suspension, which was then heated in a boiling water bath for 20 min. After cooling of the reaction mixture, residual H_2_O_2_ was decomposed by adding 20 µl of 10% Na_2_SO_3_. The mixture was then acidified with 500 µl of 6 M HCl. The resulting solution was centrifuged and an aliquot of 10 to 50 µl of the supernatant was used for each run of HPLC.

### High-performance liquid chromatography (HPLC) of oxidized melanins

The oxidation products were analyzed by HPLC using a Shimadzu LC-600 liquid chromatograph, Hamilton PRP-1 C18 column (250×4.1 mm dimensions, 7 µm particle size), and Shimadzu SPD-6AV UV detector. The mobile phase was 0.1% trifluoroacetic acid in water (solvent A) and 0.1% trifluoroacetic acid in acetonitrile (solvent B). At 1.0 mL/min, the elution gradient was (min, %B): 0, 0; 1, 0; 12, 25; 14, 25; 16, 0. The UV detector was set at a 255 nm absorbance. The standards for peak identification were pyrrole-2,3,5-tricarboxylic acid (PTCA) and pyrrole-2,3-dicarboxylic acid (PDCA).

### Synthesis and ^1^H and ^13^C NMR of HPLC standards PTCA and PDCA

Pyrrole-2,3,5-tricarboxylic acid (PTCA) was synthesized using a method adapted from [16]. First, a solution of 100 mg 5-hydroxyindole-2-carboxylic acid (Sigma) in 100 ml of 1 M K_2_CO_3_ was oxidized by adding 4 ml of 30% H_2_O_2_ and heating under reflux for 20 min. After the reaction mixture cooled to room temperature, 5 ml of 10% Na_2_SO_3_ was added to stop the oxidation reaction. The mixture was acidified with 40 ml of 6 M HCl, then extracted three times with 100 ml diethyl ether. The ether extract was washed with 50 ml water. Anhydrous Na_2_SO_4_ was added to the extract and then removed using vacuum filtration. The resulting dry ether solution was allowed to evaporate to dryness in a sidearm Erlenmeyer flask at atmospheric pressure. About 20 mg of pale brown PTCA crystals formed on the walls of the flask, and were collected for HPLC, elemental analysis and NMR. Elemental analysis: calculated for C_7_H_5_NO_6_·CH_3_COOH: C, 41.7; H, 3.5; N, 5.4%; found: C, 41.2; H, 3.0; N, 6.5%. The ^1^H NMR of PTCA showed that of the five protons in PTCA, three belong to carboxylic acid groups and are exchange averaged with the residual water whose resonance appears at about 4.5 ppm. The chemical shift of the proton at the 1 position was found to be 12.873 ppm (predicted: >12 ppm) and is exchange broadened. The known ^1^H spectrum of pyrrole carboxylic acid (from Sigma-Aldrich website) shows the proton that is bound to the nitrogen to have a wide peak at 11.7 ppm. The chemical shift of the proton at the 4 position is a doublet at 7.125/7.117 ppm (predicted: 7.3 ppm). The proton-decoupled ^13^C NMR spectrum of PTCA showed that the total number of peaks (seven) matches the total number of carbons in PTCA. The chemical shifts of each of the seven carbons were found to be (carbon #, predicted/found chemical shifts): 1, 128/126.9 ppm; 2, 117/119.4 ppm; 3, 117/118.5 ppm; 4, 128/129.8 ppm; 5, 164/161.5 ppm; 6, 172/168.2 ppm; 7, 164/161.5 ppm.

Pyrrole-2,3-dicarboxylic acid (PDCA) was synthesized using the above procedure, except with 100 mg of 5-hydroxyindole (Sigma). About 15 mg of darker brown PDCA crystals formed on the walls of the sidearm Erlenmeyer flask, and were collected for HPLC, elemental analysis and NMR. Elemental analysis: calculated for C_6_H_5_NO_4_·H_2_O: C, 41.6; H, 4.1; N, 8.1%; found: C, 40.9; H, 4.3; N, 7.3%. The ^1^H NMR of PDCA is showed that of the five protons in PDCA, two belong to carboxylic acid groups and are exchange averaged with the residual water whose resonance appears at about 4 ppm. The chemical shift of the proton at the 1 position was found to be 12.622 ppm (predicted: >12 ppm) and is exchange broadened. The signals representing the protons at the 4 and 5 positions are triplets that are centered at 6.680 and 7.085 ppm, respectively (predicted: 6.3 and 7.2 ppm). The proton-decoupled ^13^C NMR spectrum of PDCA showed that the total number of peaks (six) matches the total number of carbons in PDCA. The chemical shifts of each of the seven carbons were found to be (carbon #, predicted/found chemical shifts): 1, 123/126.0 ppm; 2, 115/118.8 ppm; 3, 110/114.2 ppm; 4, 123/124.0 ppm; 5, 172/169.8 ppm; 6, 164/161.7 ppm.

### Electron paramagnetic resonance (EPR)

EPR spectra were obtained at room temperature for each of the melanins using a Varian E112X-Band model spectrometer equipped with a TE102 resonator. Approximately 10 mg of melanin were loaded into 4 mm quartz EPR tubes (Wilmad LabGlass, Buena, N.J.), which were held in place in the resonator using a quartz finger dewar. Typical parameters used to acquire EPR spectra were as follows: modulation amplitude, 1.6 G; microwave frequency, 9.10 GHz; microwave power, 1.0 or 0.50 mW; and temperature, 297 K. All samples exhibited strong EPR signals, thus only one scan was required for each melanin. EPR spin quantification was performed using the nitroxide TEMPO (2,2,6,6-tetramethylpiperidine-1-oxyl) standards of different molar concentrations (100 µM, 500 µM, 1 mM) molecularly dispersed in polystyrene (with a density of 1.047 g/ml). EPR spectra were obtained at 300 K for these standards, each of which had a known mass of TEMPO. The melanin and standard spectra were obtained under identical instrumental conditions. Double integrals of these spectra gave the area under the curve for the absorbance spectrum in arbitrary units of amplitude. Since the molecular mass of TEMPO is 156.25 g/mol and it has 6.02×10^23^ spins/mol, TEMPO has 3.86×10^21^ spins/g, it was determined that there were 7.2×10^11^ spins/unit amplitude for a gain of 8.00×100. Spins/g quantification for each of the melanins was performed by obtaining double integrals of the EPR spectra, accounting for any difference in gain, and multiplying the result in arbitrary units of amplitude by 7.2×10^11^ spins/unit amplitude.

EPR spectra were also obtained at 77 K in samples before and after a 30,000 cGy dose from ^137^Cs (661.6 keV). The irradiation was performed with the EPR tubes at 77 K in a styrofoam box containing liquid nitrogen and the tubes were transported to the spectrometer in liquid nitrogen. Typical parameters used to acquire EPR spectra at 77 K were as follows: modulation amplitude, 1.6 G; microwave frequency, 9.11 GHz; and microwave power, 0.10 mW. Quartz EPR signal generated as a result of the irradiation was subtracted from the spectra of irradiated samples.

### Calculation of attenuation coefficients and dosimetry

Mass attenuation coefficients of melanins for the energy range of 0.1 to 200 keV were extracted from EGSnrc Monte Carlo code system [17]–[19], accounting for Rayleigh scattering, the photoelectric effect, and Compton scattering. Elemental mass percentage data obtained from elemental analysis were used to generate the simulations. This method is further described in the Supplementary Methods (Methods S1 and Fig. S1). Dosimetry and beam quality (effective energy) measurements were performed using a Farmer type ion chamber as detailed in Methods S1, Fig. S2 and S3, Tables S1-S4 [20]–[24].

### Clonogenic survival assay

Modified clonogenic survival assay [25] was performed by plating CHO cells at 16,000 cells/well into 6-well plates and incubating overnight. Synthetic melanin was added to each well to a final concentration of 20 µg/ml and allowed to incubate for 90 minutes. Controls had either no melanin or melanin ‘ghosts’ from *C. neoformans*. Plates were then irradiated with 600 cGy of 200 or 320 kV_p_ of X-ray radiation from an orthovoltage machine (effective energies of 85 or 113 keV, respectively). After irradiation, plates were washed twice with PBS, fresh medium was added, followed by 4 days of incubation. The clonogenic survival of irradiated cells was determined by crystal violet staining. For that the cells were fixed with 100% ethanol, washed with PBS, incubated with 0.5% crystal violet for 5 min, washed with tap water, and dried at room temperature. The number of colonies was estimated using two independent methods: (i) digital images followed by calibration and ImageJ intensity quantification, and (ii) color intensity measurement using a microplate reader of crystal violet extracted by ethanol from each well (see Methods S1, Fig. S4 and S5). These measurements were converted to percent survival by dividing by values from the corresponding unirradiated control plates (e.g., irradiated cells with MEL4 divided by unirradiated cells with MEL4).

## Results and Discussion

### Elemental analysis and HPLC revealed chemical and structural diversity of synthetic melanins

The elemental analysis revealed significant differences among the five synthetic melanins (Table 1). The C∶N and C∶N∶S molar ratios gave insights into the structure of the oligomer units. For example, the ∼8∶1 molar ratio of carbon to nitrogen seen in MEL1 and MEL2 was consistent with the eumelanic oligomer units of dihydroxyindole (DHI, 8∶1 C∶N) and dihydroxyindole carboxylic acid (DHICA, 9∶1 C∶N). The slightly higher C∶N ratio in MEL2 relative to MEL1 may reflect a higher proportion of DHICA relative to DHI. A recent structural model has shown that the planar stacking of tetramers of different dihydroxyindole (DHI)-related units accounts for the chemical and structural disorder to explain melanin's broad and smooth UV-vis absorbance from 200 to 700 nm [26]; therefore, not simply elemental composition, but also structural variation in DHI- and DHICA-related units could affect absorbance at higher energies. While the oligomer units of sulfur-containing melanins are commonly stated to be benzothiazine and benzothiazole [27] (which have C∶N∶S molar ratios of 8∶1∶1 and 7∶1∶1, respectively), the synthetic MEL3b and MEL4 had C∶N∶S molar ratios of 4∶1∶1 and 6∶1∶0.5, respectively. The lower relative content of carbon to nitrogen in MEL3b and MEL4 could be explained by the presence of amino groups. In MEL3b, the higher relative content of sulfur to carbon could be due to the presence of thiol groups.

**Table 1:** Elemental composition, effective atomic number and number of stable free radicals in rationally designed melanins.

Melanin type	Precursors	Method of oxidation	%C	%H	%N	%S	C∶N, C∶N∶S molar ratio	Effective atomic number (Z_eff_)	Number of stable free radicals (spins/g)
MELex5	Dopamine	Benzoyl peroxide	68	5	3	N/D	26∶01∶00	6.43	1.15×10^18^
MEL1	Dopamine	Tyrosinase	48	3	7	N/D	7.9 ∶ 1	6.92	1.26×10^18^
MEL2	L-DOPA	Tyrosinase	52	4	7	N/D	8.4 ∶ 1	6.79	9.05×10^17^
MEL3b	L-Cysteine, L-DOPA*	Tyrosinase	34	5	11	22	3.6 ∶ 1 ∶ 0.9	10.27	7.09×10^18^
MEL4	5-S-cysteinyl-L-DOPA**	Tyrosinase	46	4	9	10	5.9 ∶ 1 ∶ 0.5	8.71	2.14×10^18^
Sigma	Tyrosine	Hydrogen peroxide	48	N/D	7	N/D	8.5 ∶ 1	7.1	N/D

N/D, not determined.

* Used 3∶2 molar ratio of L-cysteine to L-DOPA.

** Used 20∶1 molar ratio of 5-S-cysteinyl-L-DOPA to L-DOPA. L-DOPA added for catalysis.

**Figure 1:**
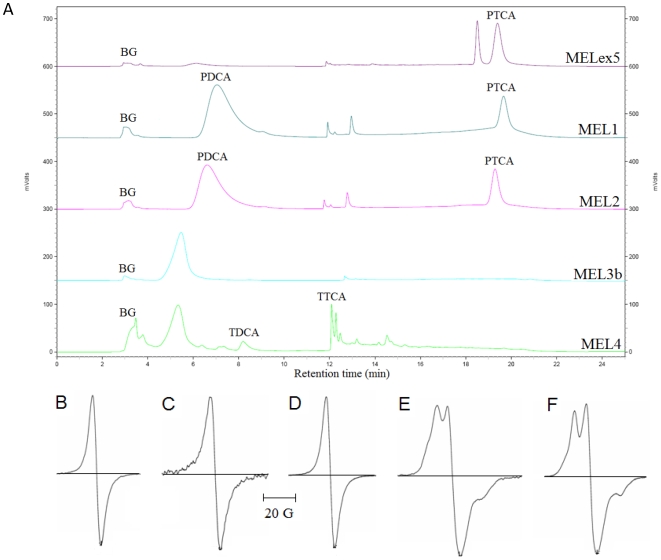
HPLC (a) and EPR spectra of (b) MELex5, (c) MEL1, (d) MEL2, (e) MEL3b, (f) MEL4. BG, background; PTCA, pyrrole-2,3,5-tricarboxylic acid; PDCA, pyrrole-2,3-dicarboxylic acid; TTCA, 1,3-thiazole-2,4,5-tricarboxylic acid; TDCA, thiazole-4,5-dicarboxylic acid.

The HPLC of oxidized synthetic melanins gave additional information about their structure (Fig. 1a). Pyrrole-2,3,5-tricarboxylic acid (PTCA) and pyrrole-2,3-dicarboxylic acid (PDCA), which are oxidation products of DHICA-derived units and DHI-derived units, respectively, were used as standards. MELex5 was characterized by a significant amount of DHICA-derived units and almost no DHI-derived units, while both MEL1 and MEL2 consisted of both DHICA and DHI-derived units, suggesting greater heterogeneity in oligomer unit composition in melanins synthesized via tyrosinase oxidation when compared to melanins synthesized with benzoyl peroxide. MEL3b, synthesized from L-DOPA with L-cysteine, did not show any oxidation products of DHICA or DHI, implying that L-DOPA does not form an appreciable amount of DHICA- or DHI-derived units when oxidized in the presence of L-cysteine. MEL4 contained no oxidation products of DHICA or DHI. However, its chromatogram had peaks assigned to 1,3-thiazole-4,5-dicarboxylic acid (TDCA) and 1,3-thiazole-2,4,5-tricarboxylic acid (TTCA), the oxidation breakdown products of sulfur-containing aromatic structures [15], [27].

### EPR studies demonstrated large stable free radical populations which were unaffected by ionizing radiation

All synthetic melanins had strong EPR signals, which is expected as EPR has been used as melanin identification test [28]–[30]. Fig. 1b–f gives the qualitative information of melanin EPR spectra. Two types of spectra were identified: those from dopamine/L-DOPA precursors (MELex5, MEL1, and MEL2) and those from sulfur-containing precursors (MEL3b and MEL4). MELex5, MEL1, and MEL2 gave single-line EPR spectra, while MEL3b and MEL4 gave more complex spectra which both had an additional peak, presumably due to hyperfine splitting of a radical centered at a ^14^N nucleus. This difference has been previously observed by Sealy *et* al [29]–[30], who compared L-DOPA melanin to 5-S-cysteinyldopa melanin and suggested the additional peak was due to a semiquinonimine radical. The spin quantification is a direct measure of the number of stable free radicals per unit mass and this quantitative information for each melanin is presented in Table 1. MEL3b and MEL4, the sulfur-containing melanins, had the highest number of spins/g, followed by MEL1, MELex5, and MEL2. Importantly, there was no change in the quantity of the stable free radicals after high-dose (30,000 cGy) ^137^Cs irradiation at 77 K (not shown), and no new radicals appeared in the spectra of irradiated melanins, indicating a high degree of radical stability as well as a robust resistance to the ionizing effects of high-energy (661.6 keV) gamma irradiation.

### Sulfur-containing melanins had higher predicted attenuation coefficients than non-sulfur-containing melanins

**Figure 2:**
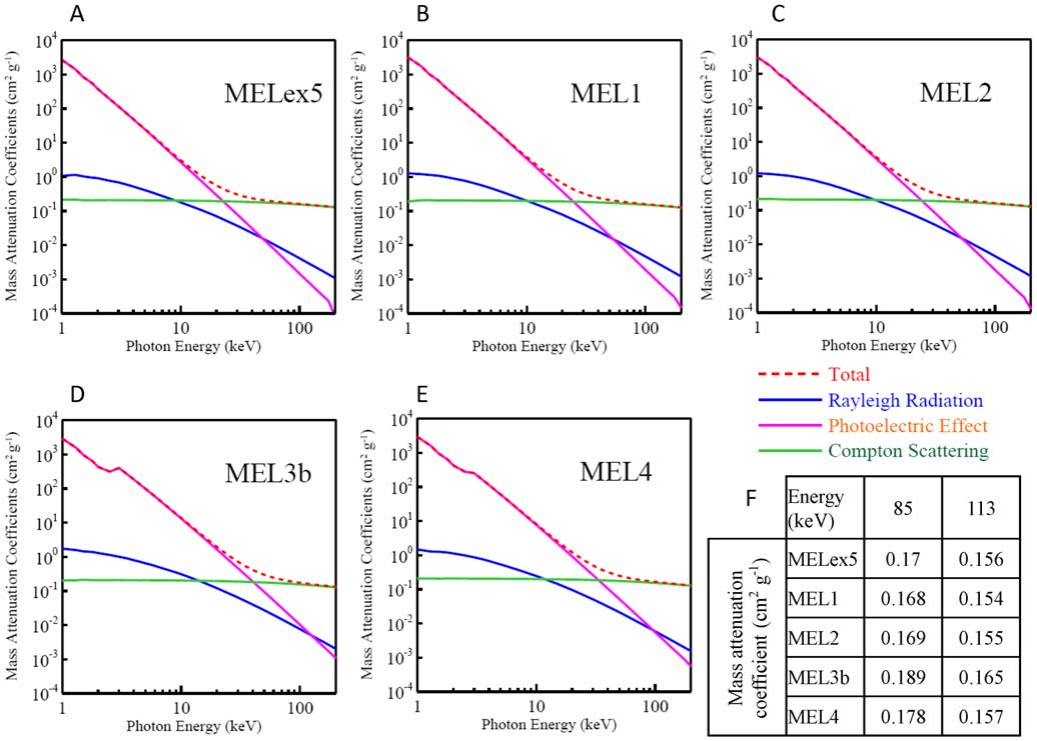
Component mass attenuation curves of (a) MELex5, (b) MEL1, (c) MEL2, (d) MEL3b, and (e) MEL4. Note that both x and y axes are logarithmic scales. Mass attenuation coefficients of interest (energies of 85 and 113 keV) are shown in (f).

The attenuation coefficient, a measure of radiation shielding properties of a material, has, to our knowledge, never been reported for melanins. Based on the elemental analysis results, we extracted the cross sections used in Monte Carlo simulations to predict the attenuation coefficients for the five melanins described above for an energy range of 0.1 to 200 keV [17]–[19]. The cross sections were tabulated as the contributions of Raleigh scattering, the photoelectric effect and Compton scattering towards total mass attenuation coefficient. The results are shown in Fig. 2 and Fig. S2. Throughout this energy range, MEL3b and MEL4 had the best shielding properties due to increased sulfur composition (the small rise in attenuation at around 3 keV is sulfur's photoelectric absorbed edge). Given that the photoelectric effect increases significantly with increasing atomic number, *Z*, (it is approximately proportional to *Z* ^3^), the presence of 10 to 20% sulfur, *Z* = 16, can result in a significant increase in energy absorption compared to the non-sulfur containing melanins. The contribution of the photoelectric effect drops rapidly with increasing photon energy, being inversely proportional to the third power of the photon energy. Therefore, at energies greater than ∼30 keV, Compton scattering becomes the dominant interaction. Compton scattering also increases with sulfur content, as there are more outer shell electrons per unit mass, and these electrons are more likely to undergo a Compton scattering event. The characterization of the Compton scattering contribution to attenuation of high energy photons by these melanins is important in supporting our hypothesis that melanin's radioprotective effect is due in part to its unique ability to dissipate the energy of generated Compton recoil electrons in a controlled fashion, thus preventing secondary ionizations and the generation of reactive oxygen species.

### Rationally designed melanins protected mammalian cells from ionizing radiation of different energies

**Figure 3:**
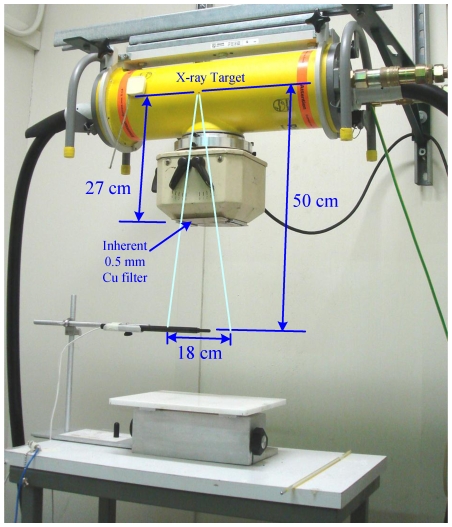
X-ray tube and experimental setup for beam calibration and mammalian cell protection studies. For cell protection studies, 6-well plates with CHO cells were placed in 18×18 cm field where the ionization chamber is located in the figure.

**Figure 4:**
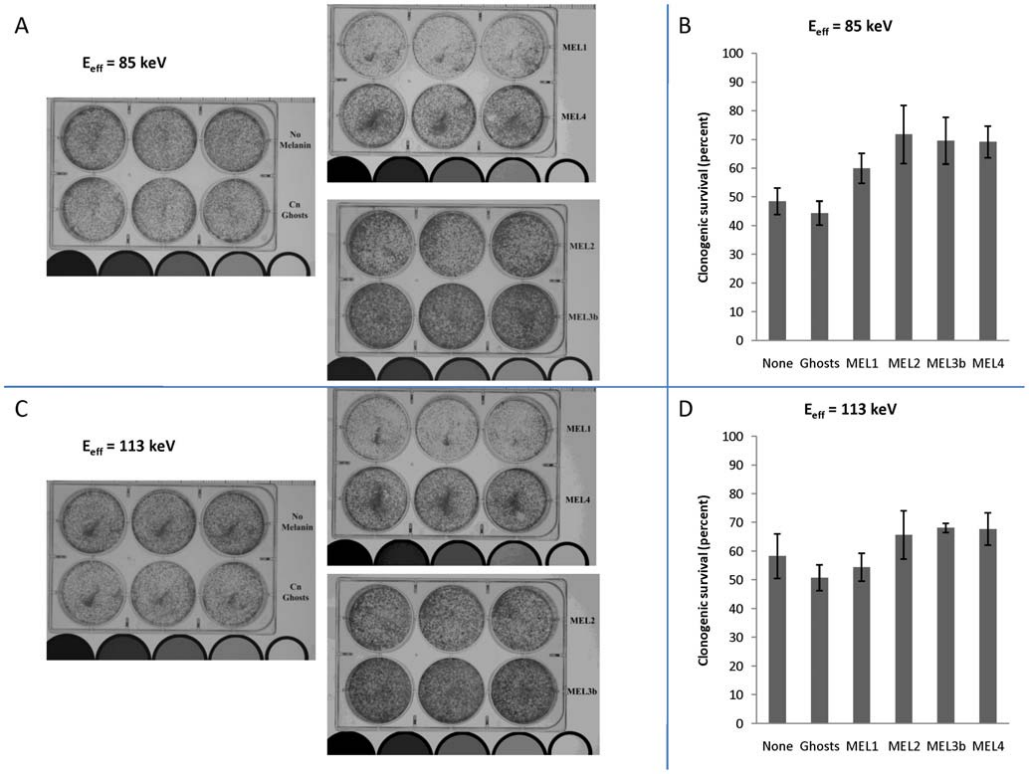
Survival of CHO cells after a 600 cGy dose with synthetic melanins compared to controls (without melanin or with melanin ‘ghosts’ from *C. neoformans*). Images of plates irradiated at (a) 85 keV (200 kV_p_) and (c) 113 keV (320 kV_p_) with each row of 3 wells corresponding to either no melanin, *C. neoformans* ghosts, MEL1, MEL4, MEL2, or MEL3b (from top left to bottom right). Clonogenic survival plots are shown for (b) 85 keV and (d) 113 keV. The clonogenic survival of irradiated cells was determined by crystal violet staining. Error bars show the standard deviations.

**Figure 5:**
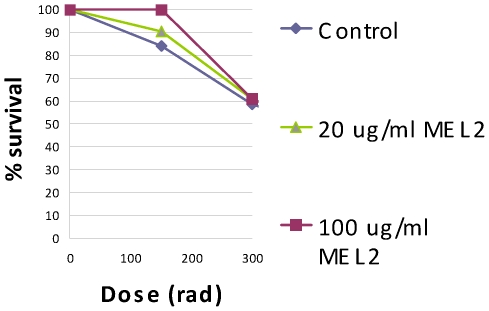
Survival of CHO cells after 661 keV ^137^Cs radiation in presence of 20 or 100 µg/mL MEL2. The clonogenic survival of irradiated cells was determined by crystal violet staining.

We then investigated the radioprotective properties of the synthetic melanins in a biological system in presence or absence of melanins by irradiating Chinese hamster ovary (CHO) cells – a cell line extensively used in radiobiology – with doses of X-rays of different energies that are lethal for humans (experimental set-up, Fig. 3). The clonogenic survival of irradiated cells was determined by crystal violet staining. To investigate whether fungal melanins conferred radioprotection of CHO cells, we also mixed CHO cells with *C. neoformans* fungal melanin shells (dubbed “ghosts” in [2]), which conferred a protective effect towards non-melanized fungal cells in our previous studies [11]. MEL2, MEL3b, and MEL4 were found to be equally radioprotective in spite of having very different numbers of stable free radicals as per EPR: CHO cells incubated with either MEL2, MEL3b, or MEL4 manifested 23, 21, and 21% increases, respectively, in survival after 600 cGy irradiation at 85 keV (200 kV_p_) when compared to controls with no melanin (P<0.05); and 7, 10, and 9% increases, respectively, in survival at 113 keV (320 kV_p_) (P<0.05) (Fig. 4). In contrast, the presence in cell culture of MEL1 – the melanin with the lowest attenuation coefficient of all melanins tested for radioprotection and with intermediate number of stable radicals - produced no dramatic increases in cell survival, providing an internal control for the association of the radioprotective effect with only certain types of melanin under given experimental conditions. The radioprotection by melanins was also dose-dependent – Fig. 5 shows the data for irradiation of CHO cells with 661 keV ^137^Cs radiation in presence of 20 or 100 µg/mL MEL2 with the higher concentration of melanin being significantly more radioprotective than the lower one.

We note that radioprotection by synthetic melanin was modest in these proof-of-principle experiments which were not designed to achieve the highest possible radioprotection but to rather demonstrate the difference between melanins with various structures with the aim of explaining the interaction between melanins and ionizing radiation. For example, the melanins used throughout the study were dried in order to determine true attenuation coefficients and numbers of paramagnetic ions. It has been shown by several research groups (reviewed in [8]) that drying of melanins changes significantly their properties, possibly, due to some structural collapse. Thus, it might be possible that radioprotective properties of hydrated melanins are higher and also that the relative inability of a particular melanin to fully hydrate under given experimental conditions might result in its lower radioprotection in spite of having the highest number of stable radicals and the highest attenuation coefficient as was observed for MEL3b. Also, the melanin in our experiments was outside the cells in a suspension form and might be much more effective as a radioprotector if it would be delivered inside the cells by nanoparticles, for example. We have recently observed the radioprotective effect of MEL4-covered nanoparticles on bone marrow in irradiated mice where nanoparticles have concentrated due to the body self-sieving effect because of their size while melanoma tumors in these mice were not protected from radiation (Schweitzer AD et al., unpublished observations).

Combining the findings of the physico-chemical and *in vitro* studies, we can make deductions on the mechanisms of radioprotection by melanins. Melanins, as any organic material, are unlikely to cause enough attenuation of high-energy photons to result in appreciable radioprotection due to ‘shielding’. However, from a radiobiological standpoint, the Compton recoil electron is responsible for the damage due to gamma irradiation [13], as it initiates the free radical chain reaction. The remarkable finding that high-dose, high-energy gamma irradiation neither affected the stable free radical population nor generated new radical species in any of the synthetic melanins suggests that melanin has the ability to dissipate the energy of Compton recoil electrons in a controlled fashion. This ability could be explained by the π-electron-rich oligomer units present in the melanins, inferred from elemental analysis and HPLC.

We conclude that radioprotection by melanins is a complex interplay of free radical scavenging and prevention of free radical generation by Compton recoil electrons through gradual recoil electron energy dissipation by the π-electron-rich pigment, until the kinetic energy of recoil electrons becomes low enough to be trapped by stable free radicals present in the pigment. We anticipate that these results could serve as the starting point for the design of nature-inspired melanin-based radioprotective materials with a broad range of applications such as powdered melanins or melanized nanoparticles suitable for internal administration, or melanin-based composite materials for radiation shielding.

## Supporting Information

Figure S1 Screen shot of the GUI of EGSnrc system running PEGS4 to create MELex5 cross section
(0.38 MB TIF)

Figure S2 Total mass attenuation coefficient curves of synthetic melanins.
(0.07 MB TIF)

Figure S3 Attenuation curve of dose rate as a function of copper thickness
(0.06 MB TIF)

Figure S4 Plate reader absorbance vs. cell number for CHO cells stained with crystal violet with trend line, best fit linear equation, and R2 value. Error bars show standard deviations.
(0.06 MB TIF)

Figure S5 Comparison of ImageJ and plate reader methods of quantifying CHO cell density four days post 137Cs irradiation of up to 1400 cGy. Error bars show standard deviations.
(0.07 MB TIF)

Methods S1 Supplementary Methods
(0.05 MB DOC)

Table S1 Correction factors for dosimetry calculations.
(0.03 MB DOC)

Table S2 Half value layer and effective X-ray energies for each tube potential.
(0.03 MB DOC)

Table S3 Dose calibration factors and the calibrated dose rate.
(0.03 MB DOC)

Table S4 Estimated standard uncertainty (1 σ) of dose to water at the phantom surface.
(0.03 MB DOC)
