# Supplementary figures and images for: Physico-Chemical Evaluation of Rationally Designed Melanins as Novel Nature-Inspired Radioprotectors
Source: PLoS One. 2009 Sep 30;4(9):e7229. doi: 10.1371/journal.pone.0007229 (PMC2749938; doi:10.1371/journal.pone.0007229)

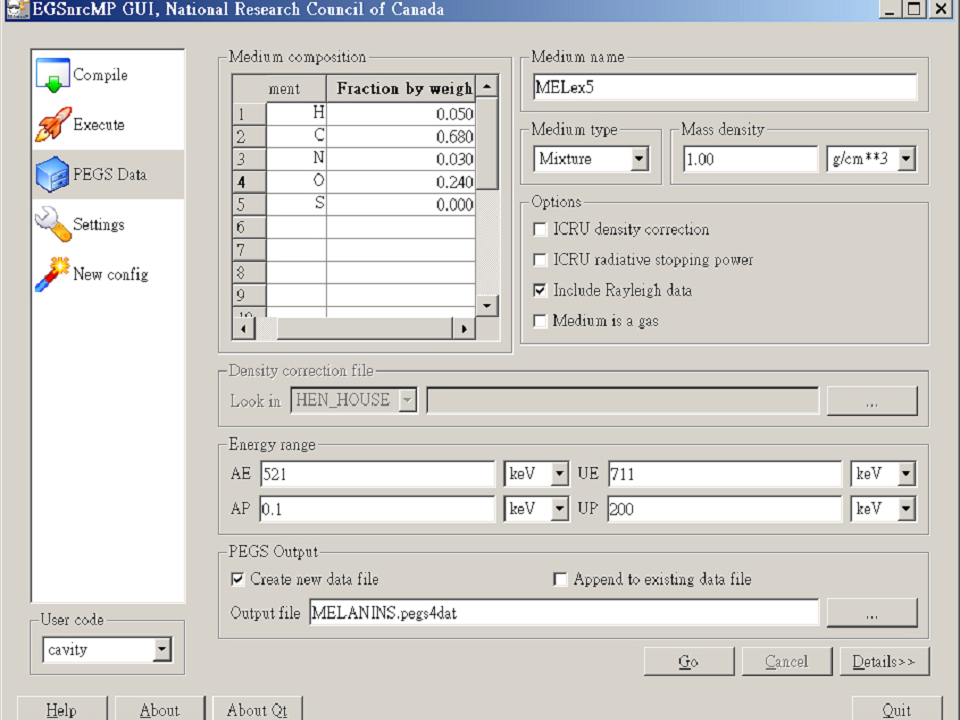

Supplement: Figure S1 — Screen shot of the GUI of EGSnrc system running PEGS4 to create MELex5 cross section (0.38 MB TIF) [file pone.0007229.s001.tif]

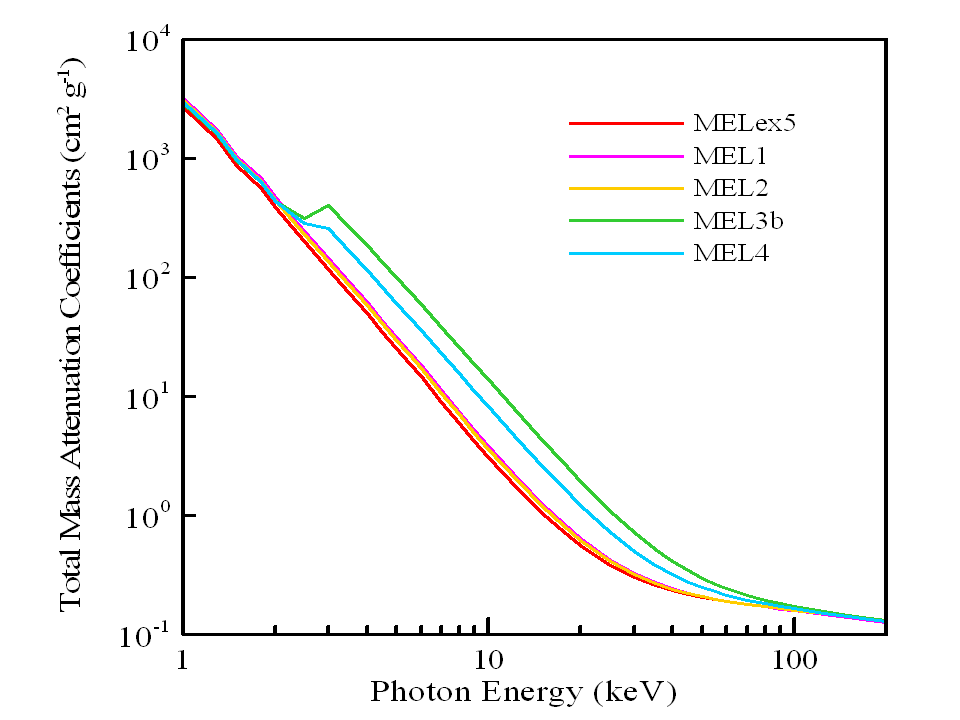

Supplement: Figure S2 — Total mass attenuation coefficient curves of synthetic melanins. (0.07 MB TIF) [file pone.0007229.s002.tif]

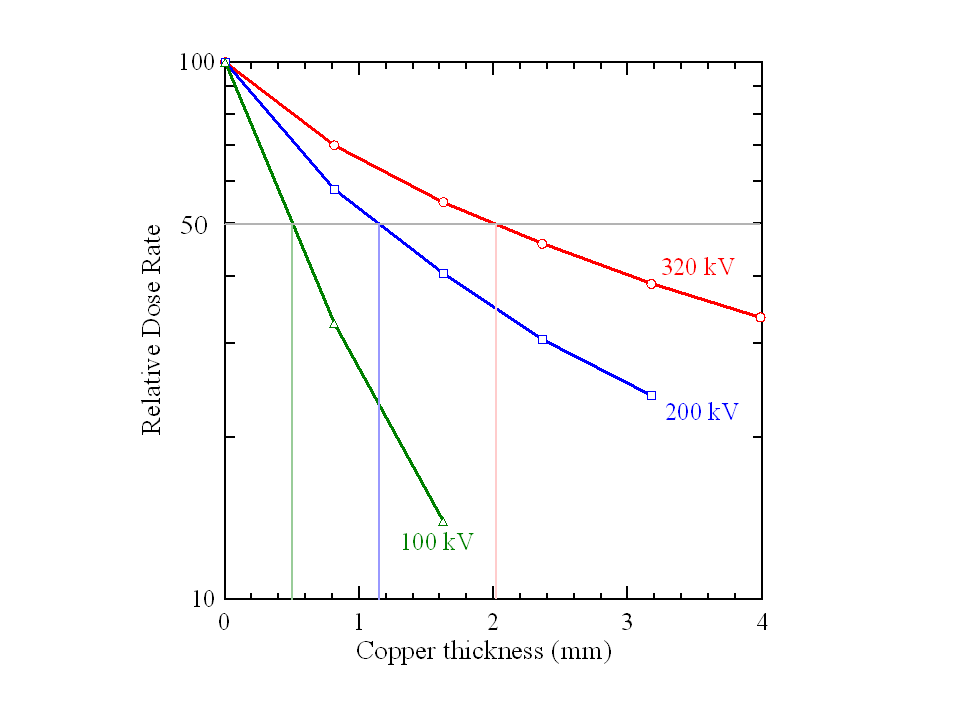

Supplement: Figure S3 — Attenuation curve of dose rate as a function of copper thickness (0.06 MB TIF) [file pone.0007229.s003.tif]

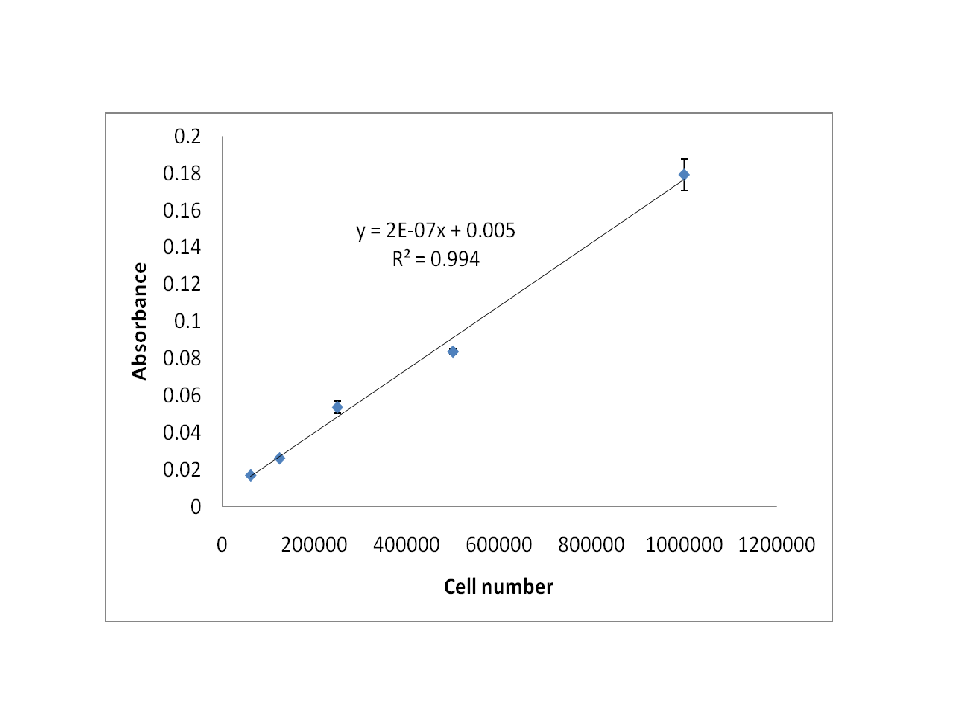

Supplement: Figure S4 — Plate reader absorbance vs. cell number for CHO cells stained with crystal violet with trend line, best fit linear equation, and R2 value. Error bars show standard deviations. (0.06 MB TIF) [file pone.0007229.s004.tif]

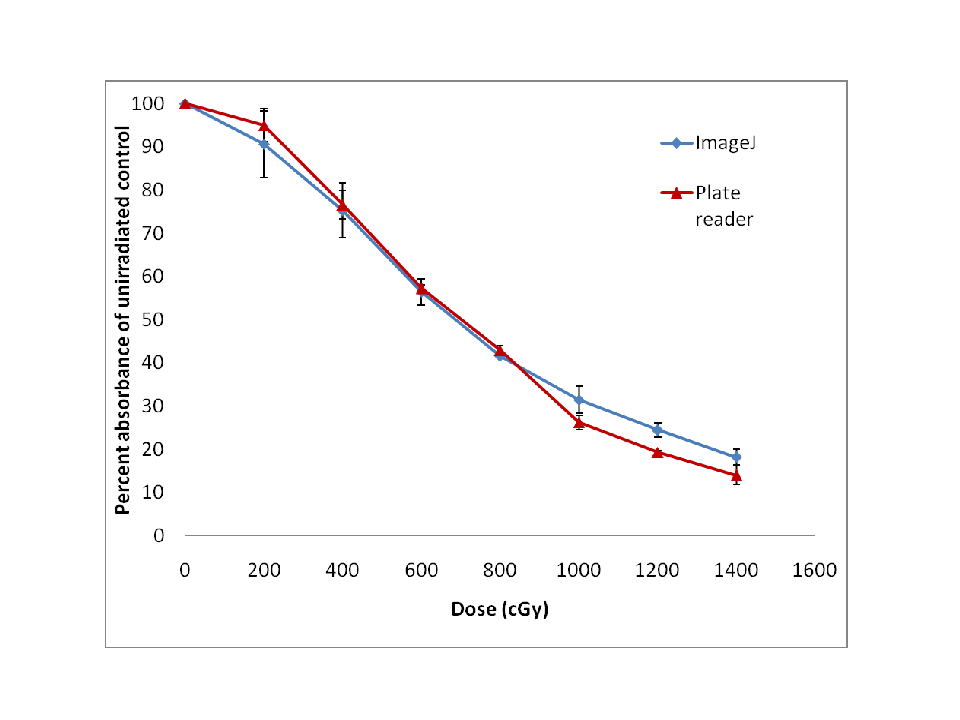

Supplement: Figure S5 — Comparison of ImageJ and plate reader methods of quantifying CHO cell density four days post 137Cs irradiation of up to 1400 cGy. Error bars show standard deviations. (0.07 MB TIF) [file pone.0007229.s005.tif]
