# Supplementary material for: Physico-Chemical Evaluation of Rationally Designed Melanins as Novel Nature-Inspired Radioprotectors
Source: PLoS One. 2009 Sep 30;4(9):e7229. doi: 10.1371/journal.pone.0007229 (PMC2749938; doi:10.1371/journal.pone.0007229)
Supplement: Methods S1 — Supplementary Methods (0.05 MB DOC) [file pone.0007229.s006.doc]

Supplementary Methods

**Mass attenuation coefficients of melanins**. Mass attenuation coefficients were calculated for the photon interaction cross section of the synthetic melanins by the PEGS4 program and extracted by the user code EXAMIN distributed with EGSnrc system, a Monte Carlo simulation code system for photon and electron transport [17-18]. The preparation program PEGS4 was used to generate the interaction cross-section for the EGS (Electron Gamma Shower) code, and the user code EXAMIN provides the function of tabulating the cross-section prepared by PEGS4.

Figure S1 shows the screen shot of the graphic user interface of EGSnrc system running the PEGS4 Data section. The melanins were treated as mixtures, and the fraction by weigh of the composite elements of H, C, N, O and S were input, respectively. The mass density was set to be 1.0 g cm^-3^ for all melanins. The total mass attenuation coefficients varied less than 0.5% when changing the input density to 0.1 or 2.0 g cm^-3^, thus the reported values of mass attenuation coefficients were found to not depend on the precise densities of the melanins, which are unknown (and measured values would be somewhat arbitrary, depending on how much the melanins are compressed). The ICRU (International Commission on Radiation Units and Measurements) density correction [19] was not used for melanins due to the lack of fitting parameters. This is not expected to affect the results, as it is known that the density effect is only relevant for the stopping power of electrons and positrons. The mass attenuation coefficients for photon energy from 0.1 keV to 200 keV were generated and exported.

**Dose rate calibrations and determination of effective energies of X-ray tube**. In order to have accurate doses for the mammalian cell protection studies, dose rates at the level of the plated CHO cells were determined as described below using a Farmer type ion chamber (Exradin A12) and an electrometer (Standard Imaging MAX 4000). Irradiations were performed using an orthovoltage machine (Philips MGC 40) with 0.5 mm copper filtration at tube potentials of 100, 200, and 320 kV_p_.

# S.1 General

A calibration procedure based on the protocol by the Radiation Therapy Committee Task Group 61 of American Association of Physics in Medicine (AAPM TG-61 protocol) for a low- and medium-energy x-ray beam (40 kV_p_ ≤ tube potential ≤ 300 kV_p_)^25^ was performed in this work.

# S.2 Dosimetry equipment and setup

The beam calibration was performed using an air-filled Farmer-typed ionization chamber (Exradin A12, Standard Imaging Inc., Middleton, WI) and electrometer (MAX 4000, Standard Imaging Inc., Middleton, WI). No buildup cap was used for the chamber wall thickness of 88.0 $\text{mg cm}^{-2}$ (50.0 $\text{mg cm}^{-2}$ for medium-energy x-ray) to provide full buildup for electron equilibrium. The measured position (source to surface distance of 50 cm) and beam field size of 18×18 cm^2^ were identical to the cell protection studies described below. Figure 3 displays the experimental setup for calibration. Notice that a copper slab of 0.5 mm was attached to the beam diagram for all irradiations. The reading of electrometer was corrected for temperature and pressure (22°C and 760 mm Hg), ion recombination, polarity effect, and electrometer accuracy. The time delay between the timer start and the achievement of desired tube potential and current, called the ‘end effect’, was measured for dose rate correction. To be consistent with cell irradiation, the end effect was measured at 7.35 mA for 100 and 200 kV_p_ tube potential, and 5 mA for 320 kV_p_ tube potential. Table S1 summarizes the correction factors.

# S.3 Beam quality

The quality of a beam depends on tube specifications such as the tube potential, target angle, target material, window material and thickness, filtration material and thickness, shape of collimation, and the source-chamber distance [20]. Referring to AAPM’s TG-61 protocol, dose calibration factor is based on the beam quality in terms of half value layer (HVL), which is defined as the thickness of an absorber of specified composition required to attenuate the intensity of the beam to half of its original value [21]. In this work, copper (alloy 101, purity of 99.99%) slabs of different thickness were used to determine the dose rate attenuated curves, shown in Figure S3. The HVLs were interpolated by the two points adjacent to 50% attenuation on semi-log plot. The effective energy of the x-ray was then calculated from the measured HVL and the mass attenuation factors listed in NIST (National Institute of Standards and Technology) x-ray database. The field size used for HVL measured was 3×3 cm^2^ to produce a narrow beam geometry [20,22]. The determined effective energies (keV) based on the HVL of each tube potential (kV_p_) are shown in Table S2.

# S.4 Dose rate determination

The in-air method suggested in AAPM’s TG-61 protocol was used to measure absorbed dose to water at the surface of a water phantom. This geometry closely approximates the absorbed dose to plated CHO cells. The dose was determined using equation (1).

$D_{w,z=0}=MN_{K}B_{W}P_{stem,air}\left[\left(\frac{\overline{\mu}_{\text{en}}}{\rho}\right)_{\text{air}}^{\text{w}}\right]_{\text{air}}$ (1)

$M$is the corrected reading from electrometer (in nC) accounting for the charge induced by the x-ray and collected from the chamber. $N_{K}$(in cGy/nC) is the air-kerma (kinetic energy released in medium) calibration factor for the given’s beam quality, which is the ratio of x ray air-kerma to the charge collected from the chamber. $N_{K}$ was adopted from the Monte Carlo calculated results of 695 ppm high-Z impurities in chamber wall published in [23]. $B_{W}$ is the backscatter factor accounting for the effect of phantom scatter, i.e., the radiation dose to the point scatter arising from interactions of the beam in the material adjacent to the measured point. In the cell protection studies, a 6-well plate with CHO cells was placed on another stand sitting on the table shown in Figure 3. The scatter dose would be caused primarily from the cell plate with dimension of 8×12 cm^2^ (equivalent diameter equal to 10.83 cm). Consequently, the$B_{W}$was then use as field size equal to the cell plate, rather than the actual radiation field. The stem correction, $P_{stem,air}$, was taken to be unity because of the large field size [24]. $\left[\left(\overline{\mu_{en}}/\rho\right)_{air}^{w}\right]_{\text{air}}$ is the ratio for water to air of the mean mass energy-absorption coefficients averaged over the incident photon spectrum. For photon beam, the dose in medium is proportional to the mass energy-absorption coefficients. Table S3 lists these parameters and the final calibrated dose rate.

# S.5 Uncertainty estimation

According to AAPM’s TG-61 protocol, the uncertainty of in-air measurement combined standard uncertainty for dose to water at the phantom surface is 3.5%. However, the realistic setup for cell irradiation differed from a perfect calibration arrangement. Table S4 listed the comparison of the uncertainty between TG-61 protocol and this work. The Monte Carlo calculation results of air-kerma calibration factor, $N_{K}$[23], due to the impurities and effective x-ray energy, ranged from 4.320 to 4.420 cGy/nC, yielding 2.3% uncertainty for the worst choice. The uncertainty of backscatter effect between semi-infinite phantom in TG-61 and cell plate in this work could be critical. The field size dependent backscatter factor increased around 10% from field diameter of 10 cm to 20 cm. The overall uncertainty would be 10.7% if the other uncertainties were identical with TG-61.

**Mammalian cell protection study**. Clonogenic assay was performed using a method adapted from [25]. Chinese hamster ovary (CHO) cells were utilized to determine percent survival under experimental compared to control conditions. Cells were harvested from a stock solution and plated at 16,000 cells/well into 6-well tissue culture treated cluster plates. Cells were incubated overnight to allow for attachment of the cells to the dishes. 20 µg/ml of each synthetic melanin was added to each experimental well and allowed to incubate for 90 minutes. Controls had either no melanin or melanin ‘ghosts’ from *C. neoformans* added. The plates were then irradiated at a dose of 600 cGy using an orthovoltage machine (Philips MGC 40) with 0.5 mm copper filtration at tube potentials of 200 or 320 kV_p_ (corresponding to effective energies of 85 and 113 keV, respectively). Dose rate calibration was completed for the conditions of this study as described above. After irradiation, all plates were washed twice with PBS, and fresh medium was added. The plates were then incubated for 4 more days. To fix and stain the cells, medium was removed, 100% ethanol was added, washed with PBS, incubated with 0.5% crystal violet for 5 min, washed with tap water, and dried at room temperature. Cell survival was estimated using two independent methods, which were verified to be excellent reflections of true cell number and were in the linear range of measurement.

The first method consisted of taking pictures using a Nikon D70 digital camera of the crystal violet-stained plates on a light source with uniform illumination. Shutter speed (1/250 s), aperture (f/9), and ASA (400) were kept constant. In order to control for the camera’s internal contrast function, neutral density filters of 0.1, 0.3, 0.6, 1.0, and 2.0. Measuring the intensity of these filters using ImageJ (NIH), we found that the camera’s internal contrast function was exponential. The neutral density filters were present in all plate pictures and were used to calibrate each image to account for automatic changes in contrast setting from image to image. Each image was split into an RGB stack and colors were inverted so that increasing darkness (which corresponds to increased cell density) resulted in an increased grayscale intensity reading. Calibration and subsequent measurements were performed using the green channel. Measured intensity values for irradiated plates were converted to percent survival by dividing by values from the corresponding un-irradiated control plates (e.g., irradiated cells with MEL4 divided by un-irradiated cells with MEL4).

The second method entailed estimating the amount of cellular crystal violet uptake in each well, which is directly proportional to cell number. This was done by adding 10 ml ethanol to each crystal violet-stained well, followed by overnight incubation to allow solubilization of crystal violet in the ethanol, then measuring the color intensity at a wavelength of 595 nm of a 1:100 dilution in a 96-well microplate using a SpectraMax 250 microplate reader. Using this method, a standard curve was generated with known cell numbers so that absorbance could be translated into an approximate cell number. As above, measured color intensity in irradiated plates was converted to percent survival by dividing by the corresponding un-irradiated control well.

Fig. S4 shows the linearity of the plate reader absorbance measurements when plotted against cell number, demonstrating that this assay is a good indicator of cell number. Fig. S5 compares the plate reader method to the ImageJ method used to quantify cell density, and demonstrates excellent agreement of these methods from 400 to 800 cGy. Therefore, these methods can be used to estimate cell survival; an index of the radioprotective effect of each melanin can thus be obtained.

One of the melanins characterized in this study (MELex5) was not fully evaluated in the CHO cell assay due to significant cytotoxicity to CHO cells in preliminary experiments.
