# Supplementary material for: Physico-Chemical Evaluation of Rationally Designed Melanins as Novel Nature-Inspired Radioprotectors
Source: PLoS One. 2009 Sep 30;4(9):e7229. doi: 10.1371/journal.pone.0007229 (PMC2749938; doi:10.1371/journal.pone.0007229)
Supplement: Table S1 — Correction factors for dosimetry calculations. (0.03 MB DOC) [file pone.0007229.s007.doc]

Table S1: Correction factors for dosimetry calculations.

| Tube Potential (kV_p_) | 100 | 200 | 320 |
| --- | --- | --- | --- |
| Electrometer Correction^1^, $P_{elec}$ | 0.999 | 0.999 | 0.999 |
| Temperature-Pressure Correction^2^, $P_{TP}$ | 1.0135 | 1.0135 | 1.0126 |
| Ion Collection Efficiency, $P_{ion}$ | 1.0007 | 1.0008 | 1.0015 |
| Polarity Correction^3^, $P_{pol}$ | 1.0167 | 1.0083 | 1.0042 |

^1^Measured by Accredited Dosimetry Calibration Laboratory, University of Wisconsin – Madison, July 19, 2006

^2^Temperature and pressure depended on the measured situation.

^3^Electrometer bias set to -302 V for final dose measurement.

^4^Corrected reading$M=M_{raw}P_{TP}P_{ion}P_{pol}P_{elec}$
