# Supplementary material for: Physico-Chemical Evaluation of Rationally Designed Melanins as Novel Nature-Inspired Radioprotectors
Source: PLoS One. 2009 Sep 30;4(9):e7229. doi: 10.1371/journal.pone.0007229 (PMC2749938; doi:10.1371/journal.pone.0007229)
Supplement: Table S2 — Half value layer and effective X-ray energies for each tube potential. (0.03 MB DOC) [file pone.0007229.s008.doc]

Table S2: Half value layer and effective X-ray energies for each tube potential.

| Tube potential (kV_p_) | 100 | 200 | 320 |
| --- | --- | --- | --- |
| Half Value Layer (mm Cu) | 0.503 | 1.145 | 2.010 |
| Effective X-ray Energy (keV) | 61.17 | 85.30 | 113.43 |
