# Supplementary material for: Physico-Chemical Evaluation of Rationally Designed Melanins as Novel Nature-Inspired Radioprotectors
Source: PLoS One. 2009 Sep 30;4(9):e7229. doi: 10.1371/journal.pone.0007229 (PMC2749938; doi:10.1371/journal.pone.0007229)
Supplement: Table S3 — Dose calibration factors and the calibrated dose rate. (0.03 MB DOC) [file pone.0007229.s009.doc]

Table S3: Dose calibration factors and the calibrated dose rate.

| Tube Potential (kV_p_) | **100** | **200** | **320** |
| --- | --- | --- | --- |
| **Current (mA)** | **7.35** | **7.35** | **5** |
| **Beam on time (min)** | **7** | **2** | **1** |
| End effect, δ_t_ | -0.0289 | -0.0320 | -0.0328 |
| Raw reading, *M_raw_* (nC) | 7.645 | 15.075 | 15.345 |
| Corrected reading, *M* (nC) | 7.875 | 15.402 | 15.611 |
| Air-kerma calibration factor, $N_{K}$ (cGy/nC) | 4.320 | 4.368 | 4.410 |
| Backscatter factor, $B_{W}$(field diameter = 10.83 cm) | 1.388 | 1.346 | 1.285 |
| Chamber stem correction factor, $P_{stem,air}$ | 1.00 | 1.00 | 1.00 |
| Mass energy-absorption coefficients, $\left[\left(\overline{\mu_{en}}/\rho\right)_{air}^{w}\right]_{\text{air}}$ | 1.050 | 1.079 | 1.089 |
| Calibrated dose rate (cGy/min) | **7.11** | **49.65** | **99.61** |
