# Supplementary material for: Physico-Chemical Evaluation of Rationally Designed Melanins as Novel Nature-Inspired Radioprotectors
Source: PLoS One. 2009 Sep 30;4(9):e7229. doi: 10.1371/journal.pone.0007229 (PMC2749938; doi:10.1371/journal.pone.0007229)
Supplement: Table S4 — Estimated standard uncertainty (1 σ) of dose to water at the phantom surface. (0.03 MB DOC) [file pone.0007229.s010.doc]

Table S4: Estimated standard uncertainty (1 ) of dose to water at the phantom surface.

| Type of quantity or procedure | % Uncertainty, TG-61 | % Uncertainty, this work |
| --- | --- | --- |
| Air-kerma calibration factor, $N_{K}$ (cGy/nC) | 0.7 | 2.3 |
| Effect of beam quality difference between calibration measurement | 2.0 | 2.0 |
| Backscatter factor, $B_{W}$ | 1.5 | 10.0 |
| Chamber stem correction factor, $P_{stem,air}$ | 1.0 | 1.0 |
| Mass energy-absorption coefficients, $\left[\left(\overline{\mu_{en}}/\rho\right)_{air}^{w}\right]_{\text{air}}$ | 1.5 | 1.5 |
| In-air measurement in the user’s beam | 1.5 | 1.5 |
| Combined standard uncertainty | 3.5 | 10.7 |
